# Osteopontin/secreted phosphoprotein-1 behaves as a molecular brake regulating the neuroinflammatory response to chronic viral infection

**DOI:** 10.1186/s12974-020-01949-4

**Published:** 2020-09-17

**Authors:** Farina J. Mahmud, Yong Du, Elizabeth Greif, Thomas Boucher, Robert F. Dannals, William B. Mathews, Martin G. Pomper, Polina Sysa-Shah, Kelly A. Metcalf Pate, Claire Lyons, Bess Carlson, Maria Chacona, Amanda M. Brown

**Affiliations:** 1 Department of Neurology, Johns Hopkins University School of Medicine, Baltimore, MD 21287 USA; 2 Department of Radiology and Radiological Science, Johns Hopkins University School of Medicine, Baltimore, MD 21287 USA; 3 Department of Molecular and Comparative Pathobiology, Johns Hopkins University School of Medicine, Baltimore, MD 21287 USA; 4 Department of Neurology and Neuroscience, Baltimore, USA

**Keywords:** Neuroimaging, Neuroinflammation, Microglial activation, DPA-713, Purkinje cells, Human immunodeficiency virus type-1, Peripheral benzodiazepine receptor, Positron emission tomography, RNAscope, Ionized calcium binding adaptor molecule 1

## Abstract

**Background:**

Osteopontin (OPN) as a secreted signaling protein is dramatically induced in response to cellular injury and neurodegeneration. Microglial inflammatory responses in the brain are tightly associated with the neuropathologic hallmarks of neurodegenerative disease, but understanding of the molecular mechanisms remains in several contexts poorly understood.

**Methods:**

Micro-positron emission tomography (PET) neuroimaging using radioligands to detect increased expression of the translocator protein (TSPO) receptor in the brain is a non-invasive tool used to track neuroinflammation in living mammals.

**Results:**

In humanized, chronically HIV-infected female mice in which OPN expression was knocked down with functional aptamers, uptake of TSPO radioligand DPA-713 was markedly upregulated in the cortex, olfactory bulb, basal forebrain, hypothalamus, and central grey matter compared to controls. Microglia immunoreactive for Iba-1 were more abundant in some HIV-infected mice, but overall, the differences were not significant between groups. TSPO^+^ microglia were readily detected by immunolabeling of post-mortem brain tissue and unexpectedly, two types of neurons also selectively stained positive for TSPO. The reactive cells were the specialized neurons of the cerebellum, Purkinje cells, and a subset of tyrosine hydroxylase-positive neurons of the substantia nigra.

**Conclusions:**

In female mice with wild-type levels of osteopontin, increased levels of TSPO ligand uptake in the brain was seen in animals with the highest levels of persistent HIV replication. In contrast, in mice with lower levels of osteopontin, the highest levels of TSPO uptake was seen, in mice with relatively low levels of persistent infection. These findings suggest that osteopontin may act as a molecular brake regulating in the brain, the inflammatory response to HIV infection.

## Background

Anti-retroviral therapy, which suppresses HIV replication to levels that impair its ability to be transmitted, has saved and allowed those in the developed world to live an average lifespan [1–3]. However, in the absence of a cure and therapy that selectively blocks HIV transcription, current treatment does not stop all immune activation and chronic inflammation. Systemic inflammation is a comorbid condition that contributes through different pathways to many chronic disorders [2]. Currently, 30–50% of HIV-infected persons experience at varying severity neuropsychiatric and cognitive impairment that significantly lowers the quality of life [4, 5] with mounting evidence suggesting that sex may play a role in differential vulnerability [6]. Deficits in learning, memory, and executive function similar to those seen in Alzheimer’s disease (AD) have been reported [7]. Unfortunately, clinical trials of anti-inflammatory agents have given conflicting and disappointing results, but studying specific AD stages has provided newer insights regarding the pathophysiology of pro- and anti-inflammatory signaling [8]. These insights demonstrate the urgent need to better understand cellular activation mechanisms and the concept of neuroinflammation in neurodegenerative disease more broadly.

HIV does not enter neurons, but rather the innate immune cells—resident microglia and macrophages in the brain become infected productively, releasing new viral progeny. The brain’s resulting injury occurs through several indirect mechanisms, which include dysregulation of glutamate metabolism and excitotoxicity, oxidative damage, and increased inflammatory signaling [9, 10]. Microglia play a central role in surveying neurons’ health and integrity and are responsible for maintaining a homeostatic CNS environment [11]. Once activated, microglia transforms from a functional resting state in which they appear highly ramified to more ameboid shapes with increased phagocytic activity and secretion of proinflammatory cytokines and chemokines activate other components of the neurovascular unit [12]. The inability of microglia to return to a basal level of homeostatic surveillance is then postulated to underlie the progression of genetic or idiopathic neurodegenerative disorders which develop over time and manifest in people at older age [13, 14].

NOD.Cg-Prkdc^scid^IL2rγ^tm1Wjl^/SzJ (NSG) immunodeficient mice reconstituted with human T cells, monocytes, and macrophages differentiated from engrafted hCD34+ hematopoietic stem cells provide the ability to study the cellular and molecular mechanisms by which HIV-induced inflammation disrupts neural functioning in the brain in a manner that is not possible in human clinical research [15–17]. Furthermore, neuroimaging, particularly positron emission tomography (PET), is a critically important non-invasive translational tool that can identify neuropathologic inflammation as a consequence of a range of injuries to the brain neurodegeneration. Indeed, despite our incomplete understanding of the neuropathophysiology, a depth of experimental evidence supports correlations between increased expression of the translocator protein (TSPO) receptor on microglia (previously known as the peripheral benzodiazepine receptor) and the extent of neuroinflammation [18]. Previous in vitro, ex vivo [19], and rhesus macaque studies [20, 21] have implicated a role for the multifunctional protein osteopontin (OPN) or secreted phosphoprotein-1 (SPP1) in proinflammatory signaling in HIV-associated neurocognitive disorders (HAND). Notably, the accumulating data points to several different underlying mechanisms; however, the detailed mechanisms of OPN function remain to be clarified. High OPN levels exist in the CSF and brains of HIV-infected persons with moderate to severe cognitive impairment [20–22]. Cortical neurons treated with recombinant OPN are protected from HIV envelope-protein-induced damage to dendrites and axons in a dose-dependent manner through mammalian target of rapamycin pathway signaling (mTORC1 and mTORC2) [23]. Interestingly, OPN is also significantly elevated in the cerebrospinal fluid of individuals with Alzheimer’s disease (AD), Parkinson’s, multiple sclerosis, and frontotemporal dementia, but very little is known about its function in the brain in these contexts (reviewed in [24]).

To test whether OPN is required or not for HIV-induced CNS inflammation, we utilized buffer-injected or HIV-infected NSG mice engrafted with human CD34^+^ cells and weekly administration of RNA-aptamers to inhibit global expression of OPN. Micro-PET-neuroimaging of female mice with the TSPO ligand [11C]DPA-713 was performed at 12 weeks post-infection. Contrary to our hypothesis, we found that microglial activation was significantly increased in the cortex, olfactory bulb, basal forebrain, hypothalamus, and central grey matter only in HIV-infected mice with decreased levels of OPN, suggesting that OPN (in female mice) functions to reduce neuroinflammation induced by HIV. Analyses of brain tissue by quantitative immunochemistry at the study endpoint demonstrated TSPO expression in Iba-1^+^ microglia and unexpectedly in specific neuronal subsets in the substantia nigra and cerebellum.

## Materials and methods

### Study design

The research objective was to test the hypothesis that osteopontin exacerbates HIV-induced neuronal injury and inflammation in the brain. NOD.Cg-Prkdc^scid^IL2rγ^tm1Wjl^/SzJ (NSG) mice were selected for this study as several publications demonstrated that this strain could be reproducibly engrafted with human immune cells and productively infected with HIV. Moreover, inflammation of the meninges and a small number of HIV-infected immune cells could be detected in the brain parenchyma [15]. With 90% power to detect a 30% difference in our experimental variables (HIV, OPN) using a two-sided test with alpha = .05, we determined that eight mice in each group would be required. NSG mice are susceptible to graft-vs-host disease and thus additional animals were included in the event that ~ 5% of mice had to be euthanized. Animals (males and females were included equally in each grouping in this study) were assigned to one of the four groups in a 2 × 2 treatment paradigm: two groups of TBS-injected animals served as uninfected controls and two groups were infected with HIV_SF162_, strain originally isolated from the brain of an HIV-infected individual with toxoplasmosis of the brain (as described below) [25]. One group of TBS-injected and one group of HIV-infected mice were intraperitoneally injected weekly with functional RNA aptamers (“OPN-R3”). The OPN-R3 aptamers blocked the interaction of osteopontin with its receptors and stimulate a feedback loop that reduces gene and protein expression [26]. The second groups of TBS- or HIV-infected animals received mutated aptamers (“mutant OPN-R3”) with sequence alterations that prevent engagement of OPN with aptamer receptors and thus served as negative controls. The animal handlers were not blinded to each group due to the biosafety level 2 requirements of working with HIV-infected mice. While we lost few mice to GVDH, we did experience losses as a result of *Corynebacterium bovis* infection (the housing rooms were *Helicobacter*-free).

### Generation of humanized mice

The animal protocol and all procedures were reviewed and approved by the Johns Hopkins University Animal Care and Use Committee and Institutional Review Board. Male and female NSG mice (5–8 weeks old) were purchased from Jackson Laboratory and maintained under pathogen-free conditions. Mice were first acclimated to the suite with automatic watering system for 2 weeks before monogamous pairing. Mice had free access to food and water and were housed in automatically controlled light conditions (light 7 am–9 pm). To avoid secondary microbial infections of these immunocompromised mice, cages were changed in a vented biosafety cabinet. All work surfaces, as well as cages, were thoroughly sanitized with chlorhexidine solution. Two-day old pups were irradiated with 100 cGy using a Gamma Cell-40 Cesium Extractor (Theratronics) followed by sedation with isoflurane in an induction chamber. The sedated pups were subjected to intrahepatic injection of 1–2 × 10^5^ cells/mouse of human CD34^+^ HSCs in 50–60 μl of sterile PBS with a 30-gauge needle (HSCs from cord blood from STEM Cell, Inc., or fetal liver from Advanced Bioscience Resources using magnetic bead separation, Miltenyi Biotec, 113-117-043). The engrafted pups were weaned at 3–4 weeks of age and males and females housed as separate groups. Peripheral blood (~ 50 μL) taken by facial bleed was sampled at regular intervals to quantify human leukocytes by seven-color flow cytometry with the following antibodies in a protocol optimized for small mouse blood volumes: human pan-CD45-Viogreen or PerCP (Miltenyi Biotec, 130-110-638, ThermoFisher MHCD4531), CD3-PE (ThermoFisher, MHCD0304), CD4-Vioblue (Miltenyi Biotec,130-113-219), CD8-APC-Vio770 (Miltenyi Biotec, 130-113-155), CD14-APC (BioLegend, 301808), CD19-PerCP (eBiosciences, 11-0199-42; BioLegend, 302228), and mouse CD45-FITC (eBiosciences,11-0451-82). Three to 4 months after engraftment, mice were sedated with isoflurane and intravenously injected with 1 × 10^5^ tissue culture infectious doses/ml of HIV_SF162_ in ~ 50 μl or with TBS buffer using a 25-gauge needle. The HIV-infected and buffer-injected mice were housed in separate same-sex social groups (when possible).

### Treatment with inhibitory aptamers

Two weeks after infection, mice were injected (intraperitoneal) with 1 mg/kg of HPLC-purified RNA aptamers against OPN (IBA Lifesciences). The sequences for the aptamers were as follows: mutant OPN-R3 (CGG-CCA-CAG-AAU-GAA-UCA-UCG-AUG-UUG-CAU-AGU-UG) and OPN-R3 (CGG-CCA-CAG-AAU-GAA-AAA-CCU-CAU-CGA-UGU-UGC-AUA-GUU-G). Both aptamers were modified at the 2′-O position with a methyl group, and the OPN-R3 aptamer included phosphorothioate modified bases (PTO) to help protect the nucleic acids from rapid attack by endo- and exonucleases (as given in reference [26]). HPLC-purified lyophilized aptamers were refolded by resuspension in Folding Buffer (1× PBS 8 mM sodium phosphate dibasic; 2 mM potassium phosphate monobasic; 137 mM NaCl; pH 7.4,) with 1 mM MgCl_2_, heated at 95 °C for 5 min (min) and allowed to cool to room temperature for 15 min before use. For the PTO modified OPN-R3 aptamer, an additional reduction step was required and performed using Tris(2-carboxyethyl)phosphine (TCEP) disulfide reducing gel slurry (ThermoScientific). A volume of TCEP slurry equal to the volume of aptamer solution was added to a microfuge tube with 3 volumes of Folding Buffer, vortexed then centrifuged for 1 min at 1000×*g* and the supernatant discarded. This step was repeated. The cooled aptamer from above was added to the washed slurry, vortexed briefly and the tube allowed to incubate at room temperature for 10 min with mixing after the first 5 min. The tube was centrifuged as above and the supernatant containing the reduced aptamer collected into a clean microfuge tube for use as described above.

### HIV viral load quantification

RNA was isolated from 56 mice using QIAamp Viral RNA mini kit (Qiagen) and concentrations determined using NanoDrop 2000 (Thermo Scientific). Samples of insufficient quality were treated with DNase to remove contaminating DNA (Life Technologies, Carlsbad, CA), followed by ethanol precipitation to concentrate any samples with less than 10 ng/μL of RNA. Samples were mixed with 1/10th volume of 3 M sodium acetate, 2.5 volumes of ice-cold 100% ethanol, and 1 μL of 20 mg/ml glycogen and kept at − 80 °C for 1 h. Samples were then centrifuged at 4 °C for 30 min, followed by two 70% ice-cold ethanol washes of the pellet. Pellets were resuspended in the appropriate volume of sterile, deionized water. RNA of insufficient concentration and quality were not used in the final results (7 out of 47 total samples). RNA was reverse transcribed in a 20 μL reaction using 50 ng/μL of random hexamers, 10 mM dNTP mix, 5X First-Strand Buffer, 0.1 M DTT, RNaseOUT recombinant RNase Inhibitor, 10 pg-5 μg of RNA, sterile water, and SuperScript III reverse transcriptase (Invitrogen/ThermoFisher). For Taqman qPCR, a custom design was optimized using forward primer Seq162F: 5′CGA-ACC-CAG-ATT-GTA-AGA-CT, reverse primer Seq162R: 5′ACA-TGC-TGT-CAT-CAT-TTC-TTC and probe: 5′-FAM/AG-CAT-TAG-G/ZEN/A-CCA-GCA-GCT-ACA-CT/3IABKFQ (Integrated DNA Technologies). Amplification was performed with TaqMan Fast Advanced Master Mix, with conditions as follows: UNG incubation 50 °C, 2 min, one cycle; enzyme activation, 95 °C, 20 s, one cycle; 40 cycles of denature 95 °C, 1 s, anneal, 48 °C, 4 s; extension, 60 °C, 16 s QuantStudio 3 (Applied Biosystems). RNA isolated from viral particles (HIV-1 _SF162_) was used to create a 10-fold dilution standard curve performed on the cDNA with a lower detection limit of 1.0 copies/ml. The slope was − 3.759 and the *R*^2^ value was 0.995, which both fell within the acceptable range for real-time PCR assays.

### Micro-positron emission tomography (PET) neuroimaging

Animals to be imaged were moved to the small animal PET imaging room at least 1 h before the experiments commenced. A dedicated small animal PET scanner (eXplore VISTA; GE Healthcare) and small animal CT scanner (X-SPECT/CT; Gamma Medica) were used. In all experiments, the PET scan was conducted for two mice simultaneously with the same batch of [^11^C]DPA-713. Batches of [11C]DPA-713 were synthesized at high specific activity and radiochemical purity at the Johns Hopkins PET Radiotracer Center according to the literature [27]. One female mouse from a pair represented a control group (Buffer-OPN^+/−^) whereas the other female mouse represented an experimental group (HIV-infected-OPN^+/−^). Mice were anesthetized with isoflurane and catheterized and PET imaging began immediately after an intravenous bolus injection of the tracer. For the quantitative analyses of uptake intensity, the following tracer parameters were recorded: mouse weight, tracer specific activity, amount of tracer injected, amount of tracer remaining in the syringe, time at which residual was measured, and start time of scan. Dynamic PET scans were acquired for 60 min (20 s × 3, 30 s × 2, 1 min × 2, 2 min × 3, 5 min × 10), and a CT scan was acquired immediately after for the purpose of localizing brain regions as described below. The individual performing the analyses were blinded to the identity of the experimental groups. PET images were reconstructed using an iterative 2D ordered-subject expectation-maximization method, using a trans-axial pixel size of 0.39 mm and axial slice thickness of 0.78 mm [28]. No attenuation and scatter corrections were applied, as they have relatively small impact on mouse brain imaging. Image analysis was performed using the PMOD software package (v3.7, PMOD Technologies Ltd, Zürich, Switzerland). For each study, the reconstructed PET and CT brain images were first co-registered; the existing Mouse Brain Template Volumes of Interest (VOIs) [29, 30] were then “morphed” to match the brain image of the fused PET-CT; finally, the pre-defined VOIs were then applied to the dynamic PET data to generate time-activity curves (TACs) in the unit of percent injected dose per gram of tissue (%ID/g).

### Tissue harvest

Mice were deeply anesthetized with isoflurane, and then subjected to intracardiac puncture for terminal blood collection followed by perfusion with cold PBS buffer. The brain was isolated from the skull intact, cut along midline in the sagittal plane into two halves, with one fixed in 4% paraformaldehyde for ~ 24 h and later transferred to 70% ethanol before further processing for paraffin embedding and sectioning into 5 μm slices (Johns Hopkins Path Services). The remaining half of brain was placed in RNALater (ThermoFisher) overnight and frozen within 18–24 h at − 80 °C before RNA extraction. RNA from the brain was isolated using Monarch Total RNA Miniprep Kit (New England Biolabs) and concentrations were determined using NanoDrop 2000 (Thermo Scientific). Each brain sample was thawed from − 80 °C and 30 mg slices were homogenized with 600 μL of 1X DNA/RNA protection reagent. Sixty microliters of Protease K reaction Buffer and 30 μL of Protease K were added, and then the tube was vortexed. Each sample was incubated at 55 °C for 7 min, followed by the addition of one volume of lysis buffer. Samples were vortexed well, and then transferred to a gDNA Removal Column. After spinning down for 30 s at 16,000 × *g*, the elute was combined with one volume of 100% ethanol and mixed by pipette. The mixture was transferred to an RNA purification column and spun for 30 s. The speed remained at 16,000 × *g* for all centrifugation steps. After the first spin in the RNA purification column, the flow through was discarded and extra RNA enhancement steps were performed as described in the Monarch Total RNA Miniprep Kit. Following these extra RNA enhancement steps, 500 μL of RNA Priming Buffer was added to the column and spun for 30 s. Two washes with RNA Wash Buffer were preformed, the second lasting for 2 min instead of 30 s. Finally, the column was transferred to a sterile 1.5 mL RNase free microfuge tube and eluted with 60 μL of nuclease free water by a 30 s spin.

### Immunohistochemistry

After warming for 10 min at 60 °C, brain sections were deparaffinized in a 100%, 95%, and 70% alcohol series with final rehydration in 1X Tris-buffered saline-TBS (0.02 M Tris-HCl, 0.15 M NaCl, pH 7.4 #351-086-101). Sections were incubated with proteinase K (Electron Microscope Sciences) at 37 °C for 20 min then immersed in citrate buffer pH 6.0 at 99 °C for 10 min then allowed to cool at room temperature for 20 min. Slides were placed in 1X TBS then treated for 10 min with Bloxall (VectorLabs) and 3% hydrogen peroxide to inactivate tissue peroxidases and alkaline phosphatases (AP). Slides were then incubated with 2% Donkey sera/0.3% Triton X-100, 0.1% Tween-20 for 1 h at room temperature, followed by blocking with Donkey anti-mouse F_ab_ (1:25; #715-007-003, Jackson ImmunoResearch Laboratories, Inc.) for 1 h. Sections were incubated with antibodies at 4 °C for at least 16 h. For double labeling, staining for TSPO was performed first followed by detection of Iba-1. Antibodies used are as follows: goat anti-mouse OPN (R&D Systems #AF808; tissue blocked in 10% rabbit serum/0.3% Triton X-100, 0.1% Tween-20), mouse anti-TSPO (1:100, MA5-24844, Invitrogen/ThermoFisher), mouse anti-Iba-1 (MA5-27726, Invitrogen/ThermoFisher), and anti-tyrosine hydroxylase (1:500, #Ab112, Abcam). Slides were rinsed in 1X TBS then incubated with 1:500 dilution of donkey anti-mouse secondary antibodies conjugated to AP (#AP15999-anti-goat, MilliporeSigma, #715-056-151-anti-mouse, Jackson ImmunoResearch Laboratories, Inc.) or horseradish peroxidase (HRP) (#715-036-150, Jackson ImmunoResearch Laboratories, Inc.). Slides were developed with the Vector Immpact Red and DAB kits (#SK-5105, #SK-4105, Vector Labs) followed by dehydration and mounting in Cytoseal 60 (#8310-4 Richard-Allan Scientific, ThermoFisher). Images were captured on a Zeiss Axio Observer A1 inverted microscope (× 20 objective), and a digital copy of the raw image was adjusted in the same manner for each, for optimal brightness and contrast, using Adobe Photoshop CS5.1. Staining intensity was quantified using ImageJ/Figi 2.0. A binary image was created and the threshold for the maximum and minimum intensity values in each channel applied. The pixel area was calculated using the measure function.

### HIV-1 RNA in situ hybridization with RNAscope

Chromogenic RNA in situ hybridization was performed using kits and protocol purchased from ACD Biosciences. HIV_SF162_ target probes were custom designed to target Env-Nef sequences obtained from the accession number M65024.1 (2621-3701). PFA-fixed paraffin embedded brains and spleens were sectioned (10 μM) and placed on SuperFrost Plus slides (Johns Hopkins Path services). Slides were baked in a slide oven for 1 h at 60 °C prior to deparaffinization. Slides were incubated twice in Histoclear for 5 min and washed in 100% ethanol twice for 1 min. Sections were incubated in Bloxall (VectorLabs) for 10 min at room temperature to quench endogenous peroxidases. Slides were then washed with distilled water and moved to a steamer containing 2 slide incubation chambers. One contained distilled water and the other contained 1X RNAscope target retrieval buffer heated to at least 99 °C. Slides were temperature adjusted by gently agitating them for 10 s in the distilled water chamber and then moved to target retrieval buffer and allowed to boil for 15 min. Slides were then washed in distilled water and transferred to 100% ethanol for 3 min and then allowed to dry. Hydrophobic barrier pen was used to make a boundary around the tissue. Proteinase K treatment was applied to the tissues and incubated in a HybEZ oven at 40 °C for 15 min. Slides were washed with distilled water and incubated with pre-warmed target probes for 2 h in the HybEZ oven at 40 °C. Slides were saved overnight in in 5X SSC buffer. Further amplification of target probe signal was performed the following day according to the manufacturer’s instructions (RNAscope 2.5 HD detection protocol). Amp 1 was applied and incubated in HybEZ at 40 °C for 30 min. Slides were washed in RNAscope wash buffer and treated with Amp 2 and incubated in HybEZ at 40 °C for 15 min. Following another wash Amp 3 was applied and incubated in HybEZ at 40 °C for 30 min. Amp 4 was applied after another wash and incubated in HybEZ at 40 °C for 15 min. Amp 5 was applied following another wash and incubated at room temperature for 30 min. Amp 6 was applied before signal detection and incubated at 15 min at room temperature. Fast red was detected by combining Red-A and Red-B (1:60) and added to the sections which developed in 10 min at room temperature. The slides were counterstained with 30% Gill’s Hematoxylin I (MilliporeSigma GHS132) and allowed to dry on a 60 °C slide warmer for 15 min. Slides were mounted with Cytoseal 60 (#8310-4 Richard-Allan Scientific, ThermoFisher) and imaged on ECHO Revolve microscope at × 40 magnification.

### Statistical analyses

Data were analyzed using ordinary one-way or grouped two-way ANOVA and subsequent Tukey’s test for multiple comparisons test (main column effect); minimum *P* < .05 was estimated as the significance level for all tests (GraphPad Prism 8).

## Results

### A model of chronic low-level HIV persistence in CD34^+^ humanized mice

**Fig. 1:**
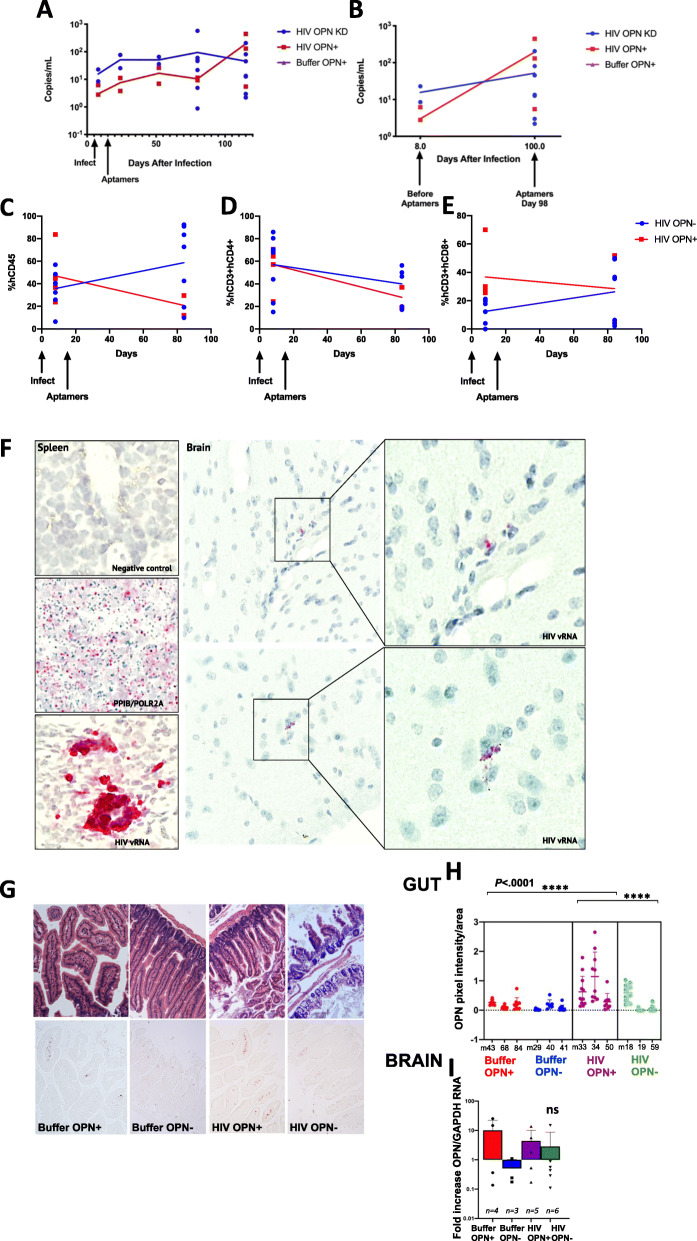
Viral load and immune cell subsets in chronically infected HIV_SF162_ humanized mice suppressed or not for osteopontin gene and protein expression. **a** Viral loads measured by Taqman qRT-PCR assay with a sensitivity of detection of 1 copy/ml, varied among individual female mice (symbols) (blue, HIV-OPN-, *n* = 8; red, HIV-OPN+, *n* = 3) in each group (simple linear regressions, no significance differences in slope, *F* = 1.471. DFn = 2, DFd = 44, *P* = 0.2408). **b** Similarly, no significant differences in viral load between the HIV OPN^+^ and HIV OPN^-^ groups before or after aptamer treatment were found (*F* = 1.329. DFn = 2, DFd = 13, *P* = 0.2983). **c**–**e** Percentage of white blood cells in plasma of HIV OPN^+^ (red) and HIV OPN^-^ (blue) female mice before HIV infection and 85 days after aptamer treatment. **c** Absolute percentage of human CD45^+^ cells (HIV OPN^+^, red, *n* = 5, start; *n* = 2 end; HIV OPN^-^ (blue, *n* = 7, start; *n* = 7 end; *F* = 3.800. DFn = 1, DFd = 17, *P* = 0.0680). **d** percentage of hCD4 in the total hCD3^+^ T cell population (HIV OPN^+^, red, *n* = 5, start; *n* = 2 end; *F* = 0.3051. DFn = 1, DFd = 17, *P* = 0.5879); HIV OPN^+^ (blue, *n* = 8, start; *n* = 6 end). **e** Percentage of hCD8^+^ in the total hCD3^+^ T cell population (HIV OPN^+^, red, *n* = 5, start; *n* = 2 end; HIV OPN^-^ (blue, *n* = 7, start; *n* = 7 end; *F* = 1.489. DFn = 1, DFd = 17, *P* = 0.2390). **f** RNAscope was used to detect HIV RNA in spleen and brain of HIV-infected mice. Panels on left (top to bottom): negative control for DapB, positive controls (green = PPIB DNA, red = POLR2A, RNA), HIV RNA in the spleen. Middle panel: HIV RNA in brain parenchyma with enlarged panels on the right showing localization to cytoplasm of cells. **g** Representative sections of gut (small intestines) stained with hematoxylin/eosin (top panels) or immunostained for OPN protein (lower panels). **h** Quantification of OPN protein immunoreactivity from female mice gut (each 10-μm section has the entire gut on the slide) subjected to neuroimaging (top graphic representation: *n* = 3 (labeled m43, etc.), 9–12 images quantified per mouse, each dot represents an image). Grouped analyses, ordinary two-way ANOVA, multiple comparisons, Tukey’s, 95% CI, *P* < .0001, ****. There was an interaction between HIV and OPN (two-way ANOVA, *F* = 6.56. DFn = 1, DFd = 104, *P* = 0.0118) and independently with HIV that accounted for 15.02% of the total variance (two-way ANOVA, *F* = 21.96. DFn = 1, DFd = 104, *P* < 0.0001) and with OPN that was responsible for 9.36% of the total variance (two-way ANOVA, *F* = 13.69. DFn = 1, DFd = 104, *P* = 0.0003). **i** Quantification of OPN mRNA levels in brain tissue of female and all (female and male mice) in each group. Ordinary one-way ANOVA, multiple comparisons, Tukey’s, 95% CI; [mean, std]: females: [*n* = 4, Buffer OPN+ 10.14, 12.19], [*n* = 3, Buffer OPN- 0.504, 0.512], [*n* = 5, HIV OPN+ 4.41, 5.71], [*n* = 6, HIV OPN- 2.86, 5.9]; all: [*n* = 7, Buffer OPN+ 12.68, 16.13], [*n* = 5, Buffer OPN- 0.363, 0.424], [*n* = 8, HIV OPN+ 6.14, 9.37], [*n* = 9, HIV OPN- 1.89, 4.89]

Despite the lack of robust HIV replication in the brain of NSG-hCD34 mice, which lack human microglia, productive replication in human target T cells and myeloid cells in the CNS and periphery induces microgliosis, astrocytosis, and alterations in neuronal metabolites [15]. We adapted this model to test the hypothesis that osteopontin (OPN) also known as secreted phosphoprotein-1 (*SPP1*) is required for HIV-mediated inflammation in the central nervous system (CNS). The experimental design included four groups composed of male and female mice engrafted with human CD34^+^ hematopoietic stem cells (hCD34, HSCs), which differentiate into mature human T cells and macrophages that permit infection with HIV. Two groups were intravenously inoculated with buffer, and the other two groups with HIV_SF162_. After 2 weeks, mice were injected weekly with either functional (HIV OPN^-^) or mutant aptamers (HIV OPN^+^) that do not block OPN expression [26]. We make note that due to (1) the capability to conduct microPET-neuroimaging on only two mice at a time, (2) the need to synthesize the radiotracer fresh each time because of its short 20-min half-life, (3) the site of tracer synthesis being located ~ 0.5 miles from the animal facility, and (4) the practicality of the microPET-neuroimager as a community resource that we could not monopolize as our own. Therefore, although male tissues were collected at the study endpoint, we had to limit the number of mice that could be subjected to microPET-neuroimaging. Given these limitations and circumstances, we decided to proceed with female mice based on a recent publication suggesting sex differences in HIV CNS disorders [31]. We recognize the limitations of not being able to include male mice in the microPET-neuroimaging studies and discuss further in the “Discussion” section. We also note the fewer data points at the earliest time periods after infection. Prior reports made no reference to the low recovery of HIV RNA from mouse blood after low temperature freeze/thaw. We discovered this fact several weeks later and subsequently performed isolations within 24–48 h. Quantitative PCR analyses of virus release into the plasma of female mice at 10 and 16 weeks after aptamer treatment revealed no significant differences between experimental groups (Fig. 1SA-B). However, analyses by two-way ANOVA demonstrated that time accounted for 20% of the variance between the viral loads at the 10- and 16-week time points (*n* = 3 HIV-OPN+, *n* = 8 HIV-OPN-, *P* = .0369). Similarly, analyses of virus release into the plasma over a period of 16 weeks (112 days) after infection revealed a wide variation in replication levels among individual female mice and overall no significant differences among the two groups were detected (Fig. 1a). However, in HIV OPN^+^ mice, replication increased from the initiation of aptamer treatment until the last sampling point at 98 days after infection, a trend that was less apparent in the HIV OPN^-^ group (Fig. 1b). During this same time period, flow cytometric analyses of circulating white blood cells in the plasma revealed a gradual and stark decline of hCD45 cells in the HIV OPN^+^, which contrasted with the higher levels detected in the HIV OPN^-^ group; however the differences in slopes were not quite significant (Fig. 1c, *F* = 3.800. DFn = 1, DFd = 17, *P* = 0.0680). While a trend in the decrease in the absolute percentage of hCD4 cells was similar in both groups, over time, the decline in hCD8 cells in the plasma of HIV OPN^-^ was less compared to the HIV OPN^+^ group, but neither trends were significant (Fig. 1d–e, CD4, *F* = 0.3051. DFn = 1, DFd = 17, *P* = 0.5879; CD8, *F* = 1.489. DFn = 1, DFd = 17, *P* = 0.2390). Therefore, we surmised that the detection of virus replication in the plasma at the 16-week study endpoint was likely due to a combination of virus release from productively infected circulating immune cells and tissue reservoirs. To detect HIV RNA (vRNA) expression in the brain and other tissues, we used in situ hybridization or RNAscope. Large foci of vRNA positive cells were readily detected in the spleen, a tissue rich in HIV target T cells and macrophage populations (Fig. 1f). In female mice with the highest viral loads, vRNA near the brain vasculature and in the parenchyma in both the infected aptamer treated groups were detected, but infrequently (Fig. 1f).

To ascertain the effectiveness of the aptamers in reducing OPN gene expression, the entire gastrointestinal (GI) tract collected at the study endpoint of female mice was immunostained for OPN protein. Because the GI tract is the largest tissue system in the body, it was used as a proxy for assessing the efficiency of the aptamer treatment. While there were no differences in OPN reactivity between the buffer treated groups, significantly elevated staining was detected in the small intestines of HIV OPN^+^ mice in contrast to infected mice injected with functional aptamers (HIV OPN^-^ group) (Fig. 1g, h). This difference in reactivity was influenced by highly significant interactions with HIV (two-way ANOVA, *P* < .0001) and OPN levels (two-way ANOVA, *P* = .0003) (Fig. 1h, right graph). Quantitative RT-PCR analyses on RNA purified from cortical brain tissue which met RNA quality standards (see Methods) showed a trend of decreased message in both the buffer OPN^-^ and HIV OPN^-^ mice treated with functional aptamers, but the differences did not reach statistical significance (Fig. 1i).

### Increased uptake of TSPO ligand in female mice with decreased osteopontin (OPN) expression

**Fig. 2:**
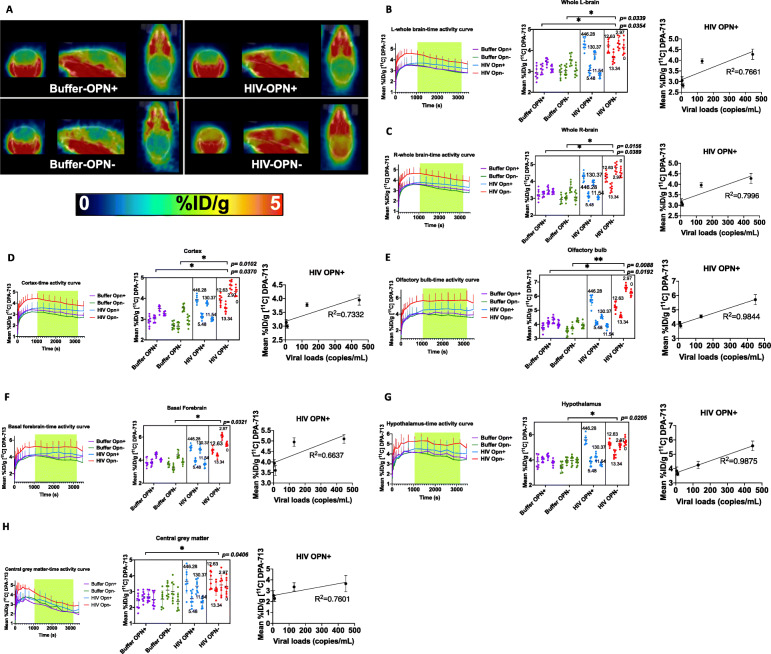

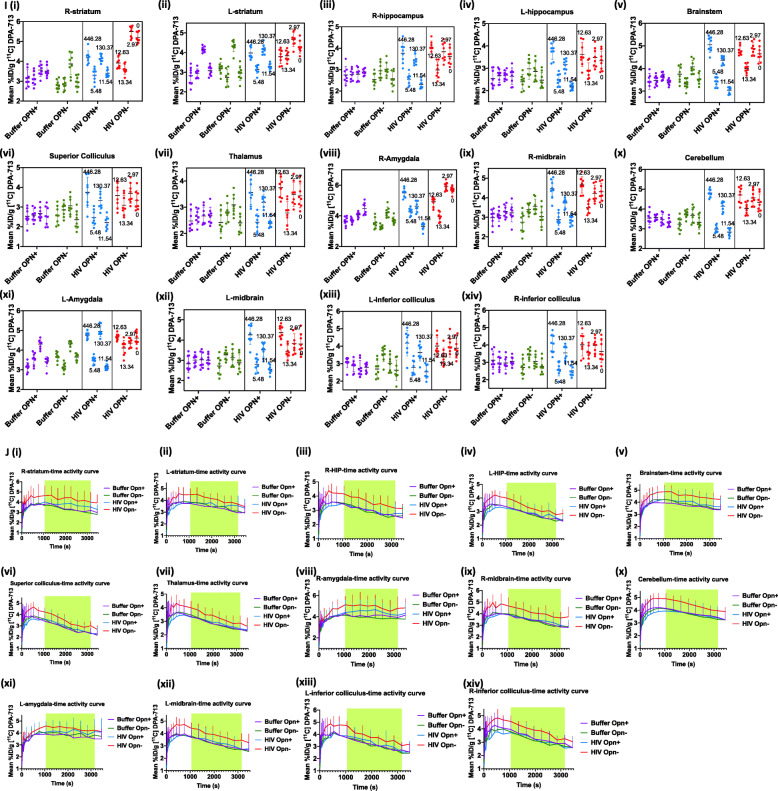
*Continued*

To determine the impact of chronic HIV infection in the presence of normal or lowered OPN levels on microglial activation in live mice, female animals in each group were anesthetized and subjected to microPET-neuroimaging using the ligand [11C]DPA-713 at 12 weeks post infection [27]. DPA-713 ligand binds to the translocator receptor (TSPO) expressed prominently on activated microglia [32] and, to a lesser extent, on the endothelium, astrocytes, and neurons of the dorsal root ganglion [33]. Significant differences in DPA-713 uptake between the group treated with functional (HIV OPN^-^) and both buffer control groups in the 20–50-min time window after bolus injection were observed across the entire brain (Fig. 2b, c, left and middle panel). Interestingly, the highest levels of uptake of DPA-713 ligand among the four experimental groups were seen in the HIV OPN^-^ group (Fig. 2b, c, left and middle panel). Analyses of specific brain regions showed significantly increased ligand uptake in the olfactory bulb, cortex, hypothalamus, central grey matter, and the basal forebrain septum of HIV OPN^-^ mice compared to the buffer control groups (Fig. 2d–h, left and middle panel). Mice in the HIV OPN^+^ group that had the highest viral loads also showed the greatest uptake of DPA-713 ligand, suggesting a direct relationship between the extent of virus replication, and the amount of TSPO binding (Fig. 2d–h, right panel). Interestingly, in the HIV OPN^-^ group, the uptake of label was the highest and sustained over time even though their viral loads were very low to undetectable, suggesting that the knockdown of OPN lead to an increase in neuroinflammation (Fig. 2d–h, left and middle panel). Analyses of other regions of the brain did not show any significant differences among the experimental groups in DPA-713 uptake (Fig. 2i, j).

### TSPO-Iba-1 immunoreactivity in cortical, hippocampal, and cerebellar regions

**Fig. 3:**
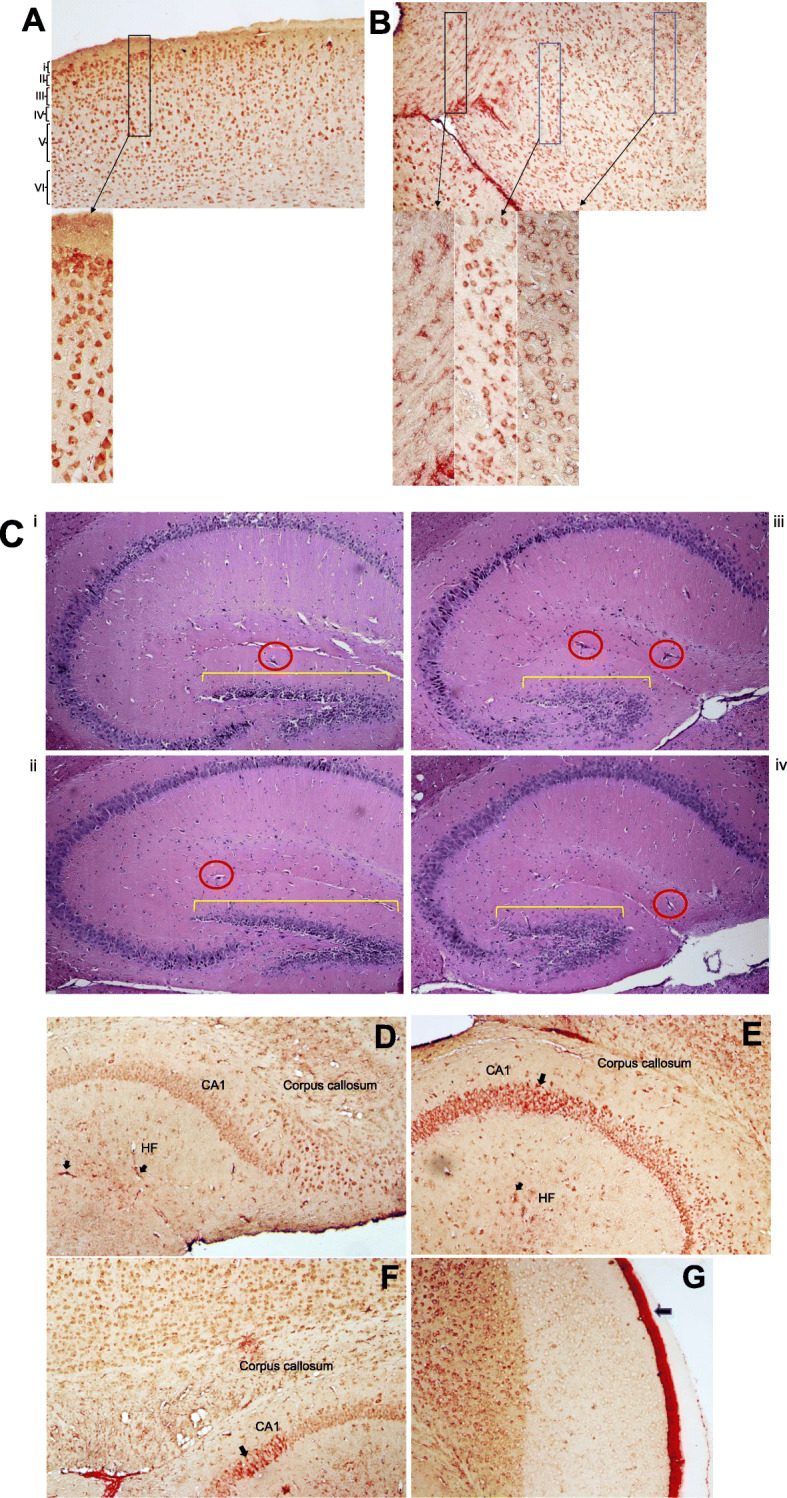
TSPO-Iba-1 double label immunoreactivity is abundant in cortical and hippocampal regions. **a**–**f** Representative samples of double-label immunostained 5 μm brain sections for TSPO (red) and Iba-1 (brown). **a** Multiple layers (i–vi) of the cortex can be seen from buffer-OPN^+^ group. **b** Midbrain region from HIV-OPN^+^ group, showing a diversity of microglia morphologies. **c** HE-stained hippocampal brain regions (i) Buffer-OPN^+^, (ii) Buffer-OPN^-^, (iii) HIV OPN^+^, (iv) HIV OPN^-^. Yellow bars, highlight the dentate gyrus and red circles indicate hippocampal fissures. **d**, **e** Hippocampus of HIV-OPN^-^. **f** Hippocampus in buffer-OPN^-^. **g** Meningeal inflammation in HIV OPN^+^

**Fig. 4:**
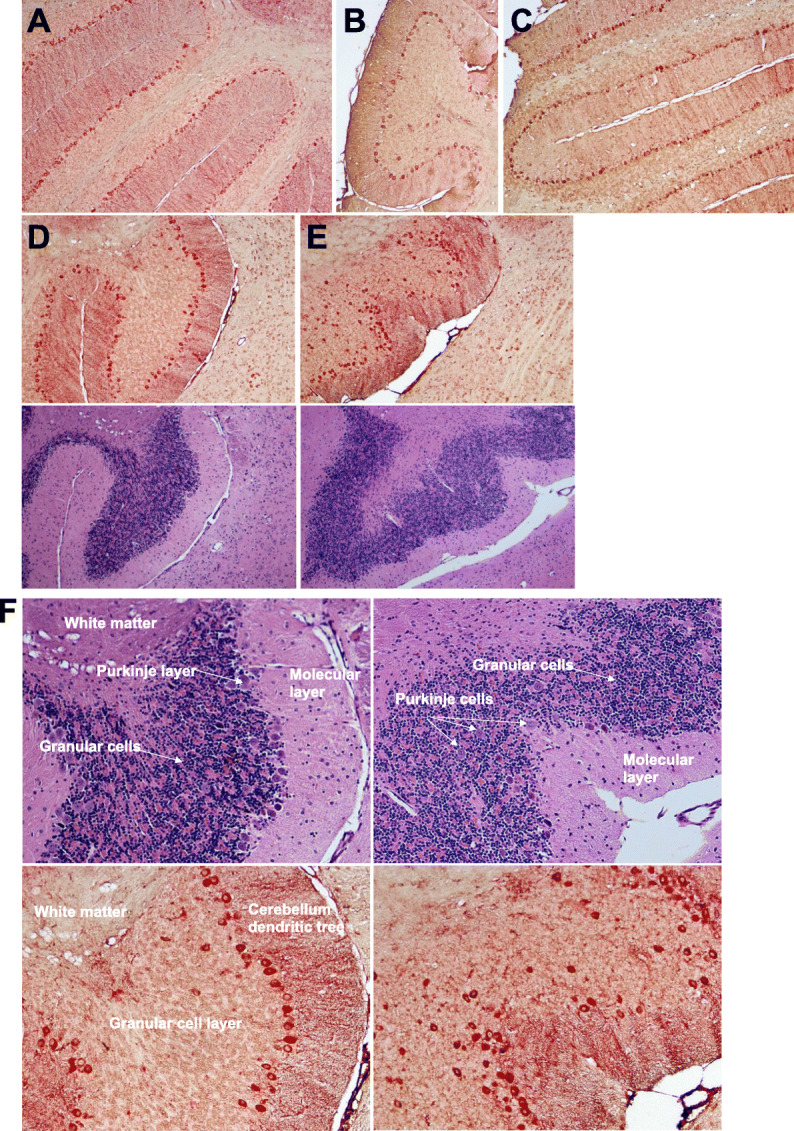
Robust TSPO immunoreactivity in a subset of neurons in the cerebellum. **a–e** Representative samples of double-label immunostained 5 μm brain sections for TSPO (red) and Iba-1 (brown) and their corresponding hematoxylin/eosin near adjacent section are shown **a** Buffer-OPN^+^, **b** HIV-OPN^+^, **c** HIV-OPN^+^, **d** Buffer-OPN^-^, and **e** HIV-OPN^-^. An ordered array of Purkinje neurons was not visible in HIV-infected mice lacking osteopontin (**e**, HIV-OPN^-^) compared to **d** HIV-OPN^+^. **f** Enlarged area from **d**, **e** is shown with the white matter, Purkinje, granular, and molecular layers of the cerebellum indicated by the arrows

**Fig. 5:**
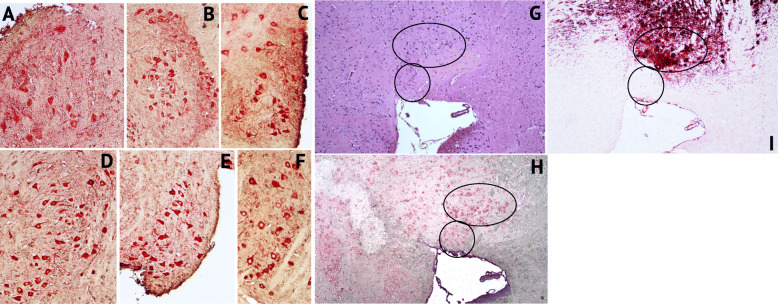
Strong TSPO immunoreactivity in a subset of tyrosine hydroxylase+ neurons in the striatum. **a–f** Representative samples of double-label immunostained 5 μm brain sections for TSPO (red) and Iba-1 (brown). **a** Buffer OPN+, **b** HIV OPN+, **c** HIV OPN+, **d** Buffer OPN-, **e** HIV OPN-, **f** Buffer OPN-. **g** hematoxylin/eosin stained section, and serial adjacent sections stained with **h** TSPO and **i** tyrosine hydroxylase. The black ovals demarcate the region of interest in each section

PET imaging with ligands that detect microglial cell activation as an indirect measure of neuroinflammation has been an active area of research as it allows insight into neuropathologic processes in living persons. However, it has been reported in unhealthy brain that cells other than microglia upregulate TSPO expression [34]. To determine whether increases in TSPO uptake detected by PET imaging in our mice correlated with measures in brain tissue at the 16-week study endpoint, double-label IHC was performed to detect cells labeling for TSPO^+^ (red color) and/or Iba-1^+^ microglia (brown color). There are relatively few published studies that have analyzed TSPO expression in brain tissue in conjunction with neuroimaging [34–37]. We first conducted a qualitative survey of brain sections from each group of female mice. Abundant TSPO reactive neurons and microglia of variable intensities were seen in the cortex (Fig. 3a, representative shown, Buffer OPN^+^). TSPO^+^Iba-I^+^ cells having ramified or dystrophic phenotypes were readily detected in the midbrain (Fig. 3b, representative shown, HIV OPN+). In both HIV-infected groups (Fig. 3c (iii and iv)), the organization of the dentate gyrus appeared altered compared to the buffer control groups (Fig. 3c (i and ii)). Additionally, there were more cells within hippocampal fissures in the HIV-OPN^+^ group compared to the other three groups (Fig. 3c (iii), red circles). TSPO labeling was strong within the hippocampal layer and fissures, and in a small number of mice, reactivity in the meningeal layer was also found (Fig. 3d–g). In contrast to the ordered array of Purkinje neurons of the cerebellum in HIV-OPN^+^ mice (Fig. 4a–c), in the HIV-OPN^-^ group, the intensely stained TSPO^+^ Purkinje neurons displayed a disordered pattern (Fig. 4e) that was not seen in the Buffer-OPN^-^ group (Fig. 4d). Moreover, a select subset of neurons, which we suspected were located in the substantia nigra, reacted very strongly with TSPO antisera (Fig. 5a–f). The identity of this brain region as the substantia nigra region was confirmed by the specific reactivity of these neurons individually with antisera against either TSPO or tyrosine hydroxylase (Fig. 5h, i).

**Fig. 6:**
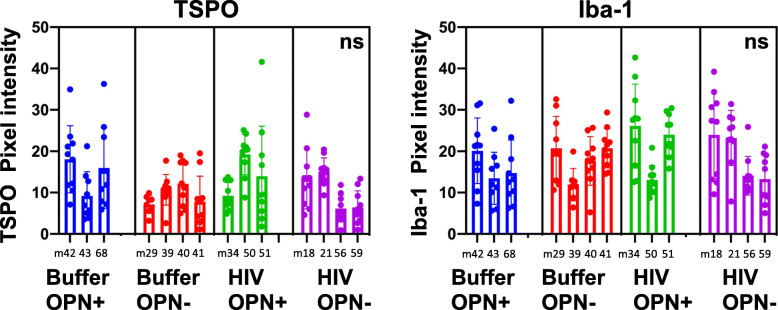
Iba-1 but not TSPO immunoreactivity is increased in the brains of mice suppressed for osteopontin. Quantitative analyses of TSPO or Iba-1 antibody reactivity, Buffer-OPN^+^ *n* = 3, Buffer-OPN^-^ *n* = 4, HIV-OPN^+^, *n* = 3; HIV-OPN^-^, *n* = 4; nested one-way ANOVA with Tukey’s for multiple comparisons. The mean and standard deviation (graphs), with significant intragroup differences (*P* < .0001, chi-square, df 17.68, 1), but no significant intergroup differences (ns) is shown

For post-mortem immunohistochemistry and quantitative analyses, brains from mice that were subjected to neuroimaging were included in the analyses (Buffer-OPN^+^, *n* = 3; Buffer-OPN^-^, *n* = 4; HIV-OPN^+^, *n* = 3; HIV-OPN^-^, *n* = 4). Quantitative analyses of TSPO antibody reactivity did not reveal any significant differences between groups (Fig. 6, TSPO), but there were detectable intra-group differences (*P* = 0.001, chi-square 14.71, 1). While there were within-group variations in the level of Iba-1 expression among HIV-infected mice, there were no significant between group differences (Fig. 6, Iba-1).

## Discussion

Knowledge that there is continuous monitoring of proper neuronal function and integrity by microglia, the resident immune cells in the CNS, fundamentally altered the way in which we think about the role these cells play in health and disease [38, 39]. Significant disruptions of the homeostatic brain microenvironment, whether secondary to injury, neurodegeneration or viral infection, dramatically activate microglia. The result is upregulation of gene expression, morphologic conversion to a functional phagocytic phenotype, and the elaboration of pro- and anti-inflammatory mediators [40]. Interestingly, recent detailed transcriptomic analyses of microglia from humans with neurodegenerative disorders, and the corresponding mouse disease models, revealed a common gene signature which includes osteopontin (OPN*/SPP1*) as a significantly enriched expressed gene in so-named neurodegenerative microglia [41–43]. Evidence that OPN is significantly elevated in the plasma and/or cerebrospinal fluid (CSF) of people with neurodegenerative disorders and in HIV-associated cognitive impairment or HAND has been reported [24]. Prior studies have examined a role for OPN in protecting neurons after injury and stroke, but few have examined its potential functional impact on microglia [44]. Our prior studies implicated OPN as an upregulated protein in HIV-infected macrophages that also stimulates viral replication through a NF-κb-dependent mechanism [22]. We went on to demonstrate, using post-mortem brain tissue from individuals with HAND, that neurons as well as microglia show elevated OPN expression [19]. OPN knockout mice are viable and display no gross physical or behavioral abnormalities but do show disorganized wound remodeling and defective macrophage infiltration after injury or infection [45, 46]. This led us to our current study to determine whether or not OPN is required for HIV-mediated neuroinflammation in the brain.

With the entry and post-entry blocks to HIV replication in mouse cells, as well as the increased genetic instability posed by mice with large numbers of transgenes, we employed the approach of functional aptamers to knockdown OPN gene expression [26]. These and closely related small nucleic acid molecules are increasingly showing promise as potential therapeutic agents that can bypass or harness the transport restrictions at the blood-brain barrier [47, 48]. We found significant suppression of OPN protein expression in the gut, demonstrating that the inhibitory aptamers had the intended effect. However, at the RNA level OPN expression showed a strong trend of lower levels in the brains of mice treated with the inhibitory aptamers, though the differences did not reach significance. Given that the average weight of a mouse brain is ~ 23 g and that we sampled 30 mg or 0.1%, it is likely that the inability to conduct comprehensive sampling limited our ability to detect differences by this method and similarly by immunochemistry for which we had only half of the brain available (split for RNA and IHC). We did conduct a survey of our mouse brain tissues for mouse OPN protein expression and saw expression in several types of cells (data not shown). As OPN protein can exist in several forms (secreted, intracellular, cleaved forms) that possess distinct biological activity, additional probing using antisera that distinguishes among these variants is needed to fully understand the dynamic interaction between aptamer inhibition and OPN expression in the periphery versus the CNS.

TSPO, formerly known as the peripheral benzodiazepine receptor, is a small 18 kDa protein found in the outer membrane of mitochondria whose expression is significantly increased in the brain after injury and neuroinflammation (reviewed in [18]); however, there is no consensus about its exact physiologic roles [18]. The availability of effective tools to clinically evaluate HIV-infected persons for neuropathologic evidence of cognitive impairment by non-invasive methods has been a critical focus of the field [32]. TSPO PET imaging with first-generation ligands showed promise in both the rhesus macaque models of HIV brain disease [49] and in humans [50]. Imaging with the second-generation ligand DPA-713 revealed significant abnormalities in the frontal cortex of HIV-infected persons with severe cognitive impairment [32]. A more recent association study found positive correlation between increased DPA-713 uptake and neurocognitive performance in treated-HIV-infected persons in line with prior human imaging studies [31, 51–53]. We believe this microPET neuroimaging study is the first of its kind performed in an HIV infectious rodent model and that despite our attention to rigor and the practical experimental limitations, as we have discussed throughout, new important insights have been learned. Future studies will be conducted to determine whether our findings are also validated or not in male mice.

The microPET neuroimaging results from this study strongly suggests that, rather than potentiating HIV injury, OPN acts as a molecular brake helping to dampen the microglial inflammatory response. In mice expressing OPN and higher levels of HIV replication, TSPO uptake was highest suggesting that in such contexts that OPN cannot fully block neuroinflammatory processes. The detection of TSPO expression in cortical, Purkinje, and striatal neurons by IHC suggests that these cells also contribute to the overall inflammatory state in the brain, but the underlying neurobiological basis for this finding is unknown [18]. In this regard, a recent report describes low-level basal expression of TSPO in several regions of the brain including vascular endothelial cells and in Purkinje neurons in normal mouse brain compared to tissues from a TSPO knockout mouse [36]. Early intraneuronal accumulation of toxic amyloid in a mouse model of Alzheimer’s disease was recently shown to initiate inflammatory gene expression of chemokines CCL2 and CCL3 in pyramidal neurons of the hippocampus, thus demonstrating that neurons have the capacity to initiate such signaling [54]. Clearly, additional studies are needed to have a more complete understanding of the neuroinflammatory response.

Moreover, TSPO can also be expressed in astrocytes. A limitation of this study is that it is microglia-centric and because of a focus on microPET neuroimaging and measures of microglia activation did not include quantification of astrocyte activation. In particular, the specialized astrocytes in the cerebellum called Bergmann glia are required for the proper development of this tissue [55]. Additionally, neurodegenerative and neuroinflammatory processes that induce death of Bergmann glia negatively impact Purkinje neuron integrity and function [56]. The TSPO labeling pattern that we observe in the cerebellum of HIV-OPN^-^ mice could be related to alterations in both Bergmann glia and Purkinje cell number, integrity, and/or activation state. The elevation of astrocyte GFAP expression as a measure of the glia inflammation in response to viral infection using different experimental models has been well-documented [57–59], although it was not detected at a significant level in a macaque model of HIV infection that found elevated TSPO binding [49]. Seminal studies revealed that astrocytes can respond in ways that exacerbate or help the brain return to homeostasis after an inflammatory process [60]. Astrocyte populations are very diverse [61] and newer markers to more faithfully detect astrocyte subtypes such as CD49f are available [62]. Conducting detailed analyses of astrocyte subtypes are central to our ongoing studies to fully understand the mechanistic interaction(s) between OPN and glial cell function in the healthy and virally infected brain.

Ruling out underlying genetic susceptibility to dementia, there are competing ideas, supported by data suggesting that cognitive deficits could be related to damage sustained early [63] or low-level chronic HIV replication in the brain [64], both of which would inflict accumulating damage to neural networks over the span of time. Our mice model in which HIV RNA expression was detected in the brain in only a few cells per five-micron section represents the latter, low-level chronic infection modeling approximately 48–55 human years. In this context, OPN expression and presumably function is required to regulate the neuroinflammatory response to HIV replication.

## Conclusions

Although OPN has diverse functions in different physiological and pathological processes, much evidence supports it having a central role in inflammatory signaling [24]. It was identified as a highly phosphorylated protein purified from osteoclasts, which are the macrophages of the bone [65, 66], but it can also be made by T cells and fibroblasts [24]. Mice express a single OPN allele. In contrast, human cells can express one or more of three splice variants of OPN, leading to an intracellular or extracellular form of protein which is further subject to proteolytic cleavage that can generate peptides possessing signaling activity (reviewed in [24]). However, the significance of these variants in regulating neuroinflammation has not, to our knowledge, been studied in the CNS. Our findings suggest that a deeper understanding of OPN regulation of innate immune signaling of neuroinflammation would be extremely valuable in the search for novel approaches which are urgently needed to promote the return to homeostasis in the CNS microenvironment in HAND and other neurocognitive disorders.

## Supplementary information

**Additional file 1: Fig. 1S**. No significant differences in viral load in chronically HIV infected humanized mice at 10 and 16-weeks post-infection. (A) (*n* = 3, HIV-OPN^+^, *n* = 8 HIV-OPN^-^, one-way ANOVA). (B) Two-way ANOVA, *F* = 5.08, DFn = 1, Dfd = 18, *P* = .0369.

## Data Availability

All data generated and analyzed during this study are included in this article and its supplementary information files.

## Abbreviations

AD: Alzheimer’s disease
CNS: Central nervous system
HAND: HIV-associated neurocognitive disorder
HIV-1: Human immunodeficiency virus type-1
IBA-1: Ionized calcium-binding adaptor molecule-1, also known as allograft inflammatory factor (AIF-1)
IHC: Immunohistochemistry
MTORC: Mammalian target of rapamycin
NSG: NOD.Cg-Prkdc^scid^IL2rγ^tm1Wjl^/SzJ
OPN: Osteopontin
PET: Positron emission tomography
SPP1: Secreted phosphoprotein-1
TSPO: Translocator protein, also known as peripheral benzodiazepine receptor (PBR)
