# Correction to: Osteopontin/secreted phosphoprotein-1 behaves as a molecular brake regulating the neuroinflammatory response to chronic viral infection

**DOI:** 10.1186/s12974-020-02002-0

**Published:** 2020-11-17

**Authors:** Farina J. Mahmud, Yong Du, Elizabeth Greif, Thomas Boucher, Robert F. Dannals, William B. Mathews, Martin G. Pomper, Polina Sysa-Shah, Kelly A. Metcalf Pate, Claire Lyons, Bess Carlson, Maria Chacona, Amanda M. Brown

**Affiliations:** 1 Department of Neurology, Johns Hopkins University School of Medicine, Baltimore, MD 21287 USA; 2 Department of Radiology and RadiologicalScience, Johns Hopkins University School of Medicine, Baltimore, MD 21287 USA; 3 Department of Molecular and Comparative Pathobiology, JohnsHopkins University School of Medicine, Baltimore, MD 21287 USA; 4 Department of Neurology and Neuroscience, Baltimore, USA

**Correction to: J Neuroinflammation 17, 273 (2020)**

**https://doi.org/10.1186/s12974-020-01949-4**

Following publication of the original article [1], the authors noticed that there are several images for Fig. 2 that are missing from the published article. Presented here is the corrected Fig. 2. Also, the original article has been updated.

**Fig. 2:**
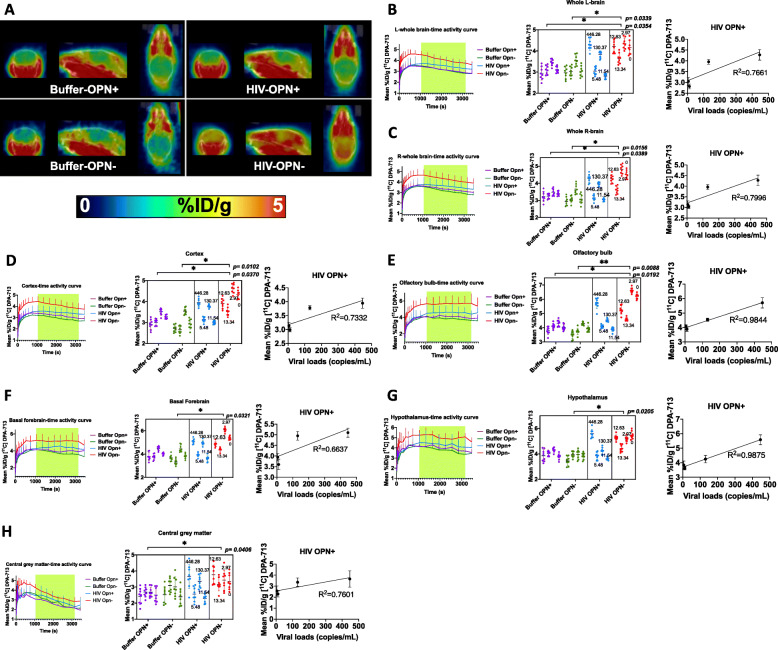

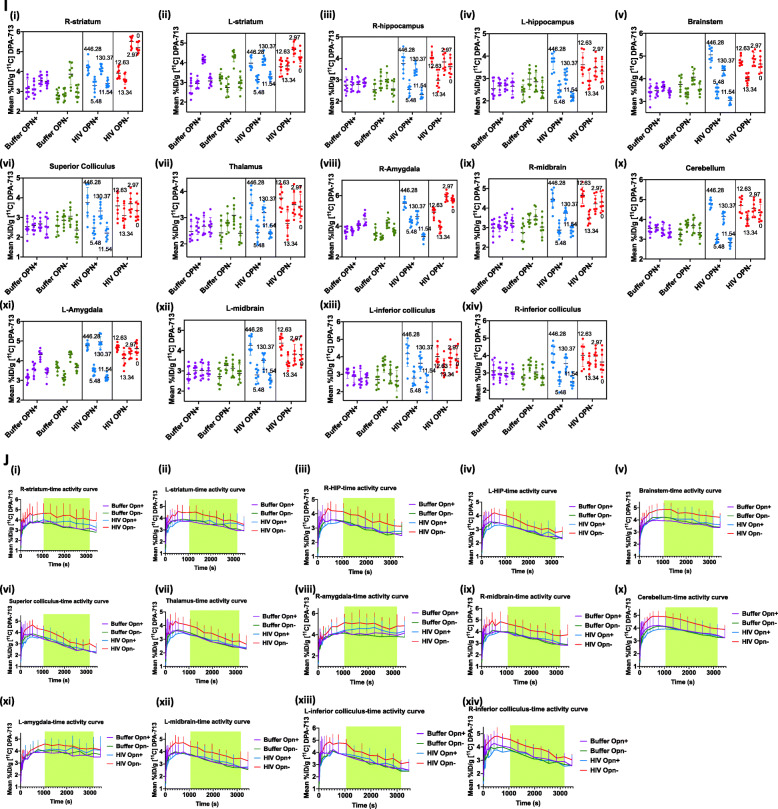
Increased uptake intensity of TSPO ligand DPA-713 in multiple brain regions in mice with global decreased expression of OPN. **a** Representative images after PET-CT reconstructed from a 45–60-min time period after injection of [11C] DPA-713 are shown. **b**–**h** Precision in sampling was increased by assessing eight independent measurements calculated over the 20–50-min uptake time period in the brain regions indicated are shown (mean pixel intensity in a region on interest (ROI). The time frame analyzed and the eight measurements calculated are shown in the brain region-specific time-activity curves (**b**–**h**, left panel, green highlighted region). The number of female mice in each group was: (*n* = 4, Buffer-OPN^+^, *n* = 4, Buffer-OPN^−^, *n* = 4, HIV-OPN^+^, *n* = 4, HIV-OPN^+^). The eight measurements from each mouse are shown as individual dots in the nested graphs (**b**–**h**, middle panel). Each group is indicated with a different color. The numbers on the HIV OPN+ and HIV OPN- section are the viral loads in copies/mL of each mouse. The data were analyzed using nested one-way ANOVA with Tukey’s multiple comparisons, with mean and standard deviation, and significance. The right panel in **b**–**h** shows linear regression between viral loads and TSPO uptake from the HIV-OPN+ group. **i**, **j** Other brain regions analyzed in the same manner as described and no significant differences between groups were detected
