# Supplementary figures and images for: Osteopontin/secreted phosphoprotein-1 behaves as a molecular brake regulating the neuroinflammatory response to chronic viral infection
Source: J Neuroinflammation. 2020 Sep 17;17:273. doi: 10.1186/s12974-020-01949-4 (PMC7499959; doi:10.1186/s12974-020-01949-4)

A

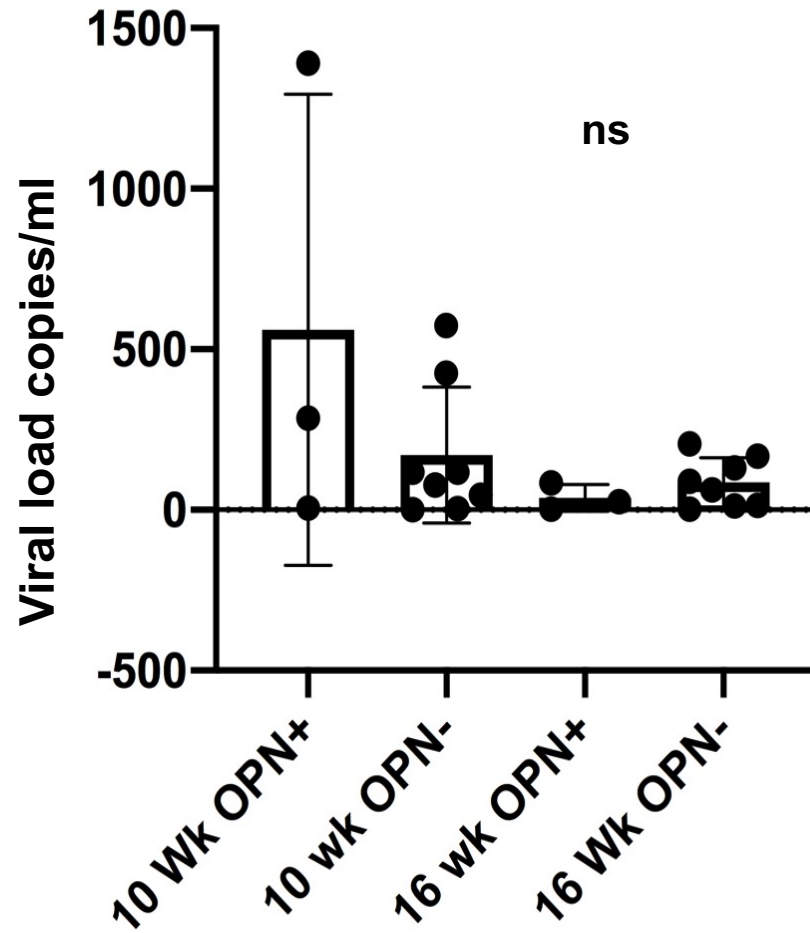

B

## 95% Confidence Intervals (Tukey)

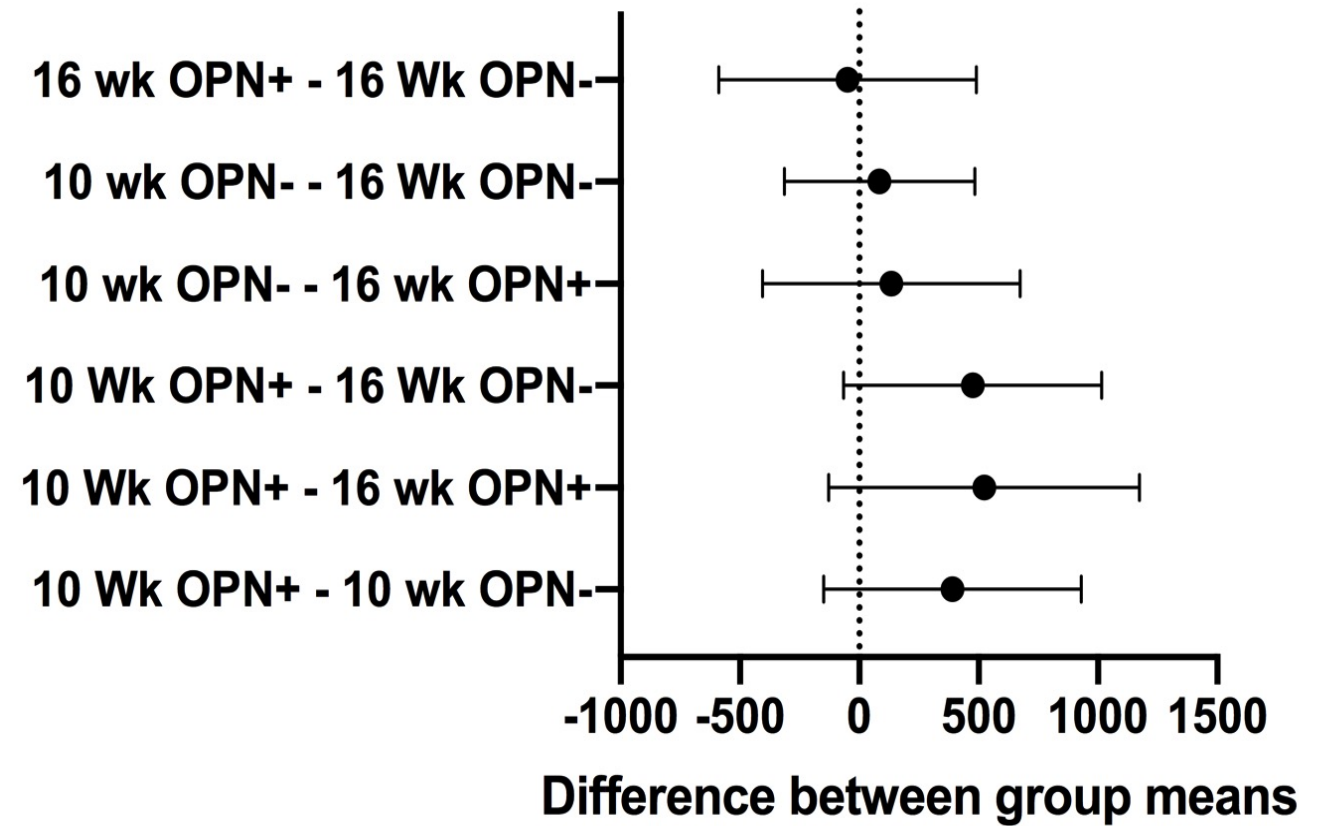

Supplement: Supplementary file 1 — Additional file 1: Fig. 1S. No significant differences in viral load in chronically HIV infected humanized mice at 10 and 16-weeks post-infection. (A) (n = 3, HIV-OPN+, n = 8 HIV-OPN-, one-way ANOVA). (B) Two-way ANOVA, F = 5.08, DFn = 1, Dfd = 18, P = .0369. [file 12974_2020_1949_MOESM1_ESM.pdf]
